# Temporary FDA Advisory Committee Members, Recommendations, and Agency Actions, 2017-2021

**DOI:** 10.1001/jamanetworkopen.2024.36789

**Published:** 2024-10-01

**Authors:** Nikhil Chaudhry, Audrey D. Zhang, Jason L. Schwartz, Reshma Ramachandran, Joseph S. Ross

**Affiliations:** 1 Collaboration for Regulatory Rigor, Integrity, and Transparency, Yale School of Medicine, New Haven, Connecticut; 2 Department of Medicine, Duke University School of Medicine, Durham, North Carolina; 3 Department of Health Policy and Management, Yale School of Public Health, New Haven, Connecticut; 4 Section of General Internal Medicine, Department of Internal Medicine, Yale School of Medicine, New Haven, Connecticut

## Abstract

This cross-sectional study examines the recommendations and agency actions of temporary and permanent US Food and Drug Administration advisory committee members from 2017 to 2021.

## Introduction

The US Food and Drug Administration (FDA) convenes advisory committees to provide expert outside counsel on key agency decisions, such as whether to approve new drugs or issue safety warnings.^1^ Typically, advisory committees consist of permanent members, who are generally appointed for multiple year terms, and temporary voting members, who participate in specific meetings based on salient expertise.^2^ However, little is known about the role temporary advisory committee members play in regulatory decision-making. Accordingly, we examined temporary and permanent advisory committee member participation, voting recommendations, and concordance with subsequent FDA regulatory actions.

## Methods

In accordance with the Common Rule, this cross-sectional study was exempt from ethics review and informed consent because it used public, nonidentifiable data and was not human participant research. We followed the Strengthening the Reporting of Observational Studies in Epidemiology (STROBE) reporting guideline.

We searched the advisory committees section of the FDA website and identified meetings convened by the Center for Drug Evaluation and Research from 2017 to 2021 that evaluated original and supplemental indication approvals or safety actions, consistent with prior research,^3^ treating each product discussed and voted upon as a separate meeting. We excluded 18 advisory committee meetings that were convened to provide general guidance, 7 that considered other regulatory actions, 15 that lacked publicly available transcripts, and 4 that did not use dichotomous voting questions.

Using meeting transcripts and member rosters, we recorded committee members’ status and vote, categorized as favoring or not favoring market availability, consistent with prior research.^3^ We used the Drugs@FDA database to determine whether the recommended action—approval, label change, or withdrawal—occurred within 12 months of the meeting. Subsequent FDA actions were considered concordant with the committee’s recommendation if the action aligned with the advisory committee’s majority vote on the single voting question (tie votes were considered nonconcordant). We used a χ^2^ test and an exact test of agreement to compare permanent and temporary advisory committee members’ recommendations concordance with subsequent FDA actions. Data analyses were performed from April to May 2024 using JMP Pro version 17.0 (JMP Statistical Software). Statistical significance was set at *P* < .05, and tests were 2-sided.

## Results

From 2017 to 2021, 88 advisory committee meetings addressing drug approval or safety actions were convened among 14 therapeutic area divisions. Overall, 1315 individuals participated in these 88 meetings, 657 (49.8%) of whom were permanent members and 658 (50.2%) temporary members (Table 1). The median (IQR) number of advisory committee members participating was 15 (12-17) and the median (IQR) proportion of temporary members on an advisory committee was 50.0% (41.7%-57.5%).

**Table 1.** US Food and Drug Administration Advisory Committee Meetings and Voting Members, 2017-2021

Year	Members, No. (%)		
	Advisory committees	Voting members	
		Temporary	Permanent
2017	20 (22.7)	154 (51.2)	147 (48.8)
2018	24 (27.3)	200 (51.5)	188 (48.5)
2019	21 (23.9)	153 (48.4)	163 (51.6)
2020	8 (9.1)	51 (45.5)	61 (54.5)
2021	35 (39.8)	100 (50.5)	98 (49.5)
Total	88 (100)	658 (50.2)	657 (49.8)

Among the 88 committee meetings, the majority vote recommended market availability (product approval or no safety label change) for 61 (69.3%), against market availability for 24 (27.3%), and votes were tied for 3 (3.4%). Overall, 75 advisory committee recommendations (85.2%) were concordant with subsequent FDA actions. When stratified by committee member status, 77 temporary member recommendations (87.5%) and 71 permanent member recommendations (80.7%) were concordant with subsequent FDA actions (risk ratio, 1.08; 95% CI, 0.95-1.23; *P* = .22) (Table 2). However, patterns of concordance differed significantly, as 9 of 11 nonconcordant temporary member recommendations (81.8%) and 10 of 17 nonconcordant permanent member recommendations (58.8%) favored market availability (*P* = .03) (Table 2).

**Table 2.** Temporary and Permanent Advisory Committee Members’ Recommendations Concordance With Subsequent FDA Actions, 2017-2021

Permanent member recommendation-FDA action	Temporary member recommendation-FDA action, No. (%)		
	Concordant	Nonconcordant	Total
Concordant	65 (73.9)	6 (6.8)^a^	71 (80.7)
Nonconcordant	12 (13.6)^b^	5 (5.7)^c^	17 (19.3)
Total	77 (87.5)	11 (12.5)	88 (100)

Abbreviation: FDA, US Food and Drug Administration.

^a^ For the 6 advisory committees where only temporary member recommendations were nonconcordant with subsequent FDA actions, 2 were recommendations to approve a new drug or indication not followed by FDA approval, 1 was a recommendation not to approve a new drug or indication followed by FDA approval, and 3 were recommendations not to issue a safety label change followed by an FDA safety action (5 of 6 favored market availability).

^b^ For the 12 advisory committees where only permanent member recommendations were nonconcordant with subsequent FDA actions, 5 were recommendations to approve a new drug or indication not followed by FDA approval and 7 were recommendations not to approve a new drug or indication followed by FDA approval (5 of 12 favored market availability).

^c^ For the 5 advisory committees where both temporary and permanent member recommendations were nonconcordant with subsequent FDA actions, 3 were recommendations to approve a new drug or indication not followed by FDA approval, 1 was a recommendation not to issue a safety label change followed by an FDA safety action, and 1 was a recommendation to issue a safety label change not followed by an FDA safety action (4 of 5 favored market availability).

## Discussion

In this cross-sectional study of drug-related FDA advisory committees from 2017 to 2021, we found temporary members made up half of all advisory committee members. While 85% of advisory committee recommendations were concordant with subsequent FDA actions, consistent with prior research,^3,4^ permanent and temporary member recommendations differed in patterns of concordance, as temporary member recommendations that were not concordant with subsequent FDA actions more commonly favored drugs’ market availability. This study offers new insights into the role temporary advisory committee members play in regulatory decision-making.

Our study was limited to drug-related advisory committees and by the inability to account for individuals participating in multiple advisory committees, differences in relevant expertise between permanent and temporary members, and whether or how committee members may influence voting. Future work could explore why temporary members are so frequently selected and how they are identified, which could inform FDA efforts to optimize advisory committees.^5^

## Notes

Supplement.
Data Sharing Statement
