# Supplementary material for: Temporary FDA Advisory Committee Members, Recommendations, and Agency Actions, 2017-2021
Source: JAMA Netw Open. 2024 Oct 1;7(10):e2436789. doi: 10.1001/jamanetworkopen.2024.36789 (PMC11445682; doi:10.1001/jamanetworkopen.2024.36789)
Supplement: Supplement. — Data Sharing Statement [file jamanetwopen-e2436789-s001.pdf]

Data Sharing Statement

Data

Data available: Yes
Data types: Data (not involving human participants)
How to access data: request for data must be sent to an individual: joseph.ross@yale.edu
When available: With publication

Supporting Documents

Document types: None

Additional Information

Who can access the data: anyone requesting the data
Types of analyses: for research purposes
Mechanisms of data availability: without investigator support
